# The evolution of shame and its display

**DOI:** 10.1017/ehs.2022.43

**Published:** 2022-10-06

**Authors:** Mitchell Landers, Daniel Sznycer

**Affiliations:** 1 Center for Early Childhood Research, Department of Psychology, The University of Chicago, Chicago, IL, USA; 2 Oklahoma Center for Evolutionary Analysis, Department of Psychology, Oklahoma State University, Stillwater, OK, USA

**Keywords:** Shame, emotion, evolution, display, camouflage

## Abstract

The shame system appears to be natural selection's solution to the adaptive problem of information-triggered reputational damage. Over evolutionary time, this problem would have led to a coordinated set of adaptations – the shame system – designed to minimise the spread of negative information about the self and the likelihood and costs of being socially devalued by others. This *information threat theory of shame* can account for much of what we know about shame and generate precise predictions. Here, we analyse the behavioural configuration that people adopt stereotypically when ashamed – slumped posture, downward head tilt, gaze avoidance, inhibition of speech – in light of shame's hypothesised function. This behavioural configuration may have differentially favoured its own replication by (a) hampering the transfer of information (e.g. diminishing audiences’ tendency to attend to or encode identifying information – shame *camouflage*) and/or (b) evoking less severe devaluative responses from audiences (shame *display*). The shame display hypothesis has received considerable attention and empirical support, whereas the shame camouflage hypothesis has to our knowledge not been advanced or tested. We elaborate on this hypothesis and suggest directions for future research on the shame pose.

## Graphical abstract

**Figure:**
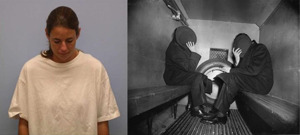

**Social media summary:** The shame display functions to appease others. Landers and Sznycer propose an additional function: to render us invisible.

## Introduction: Shame is an adaptation

Shame occurs in every known culture (Brown, 1991; Darwin, 1872/2009; Fessler, 1999); its components develop reliably and early (Barrett, 1998; Lewis, Alessandri, & Sullivan, 1992; Stipek, Recchia, & McClintic, 1992), even in congenitally blind individuals (Tracy & Matsumoto, 2008). Explicit instruction and reinforcement are unlikely to play a causal role in its development, since conditioning of social fear is resistant to extinction (Dimberg & Öhman, 1983; Öhman & Dimberg, 1978). And although there is cultural variation in its elicitors and behavioural outputs (Benedict, 1946; Fessler, 2004; Keltner & Anderson, 2000; Mosquera, Manstead, & Fischer, 2002; Shweder, 2003), shame has a culturally invariant cognitive substrate (Fessler, 1999; Scherer & Wallbott, 1994). Shame thus has the hallmarks of an adaptation.

Adaptations are packages of design features retained over evolutionary time because they reliably solved tasks tributary to reproduction in ancestral environments. An adaptationist approach to understanding shame thus begins from theories of the adaptive problems our ancestors faced over deep evolutionary time (Tooby & Cosmides, 1992, 2015). In what follows, we provide an overview of the class of adaptive problems the human shame system appears designed to solve, how shame solves such problems and how this account of shame fits with the extant literature on shame and makes novel, unique predictions. Then, we review what is known about the stereotypical human shame display and analyse this display given shame's hypothesised function.

## The adaptive problem of shame: Information-triggered devaluation

Our hominin ancestors evolved in environments characterised by high rates of mortality (Burger, Baudisch, & Vaupel, 2012), resource scarcity (Hill & Hurtado, 1996), variance in food acquisition (Hill & Hurtado, 1996; Kaplan & Hill, 1985), disease and injury (Sugiyama, 2004), and aggression from predators and conspecifics (Keeley, 1997). In contrast to other animals, including our primate cousins, humans often relied on other members of their groups for the assistance necessary to survive and reproduce (Clutton-Brock, 2009; Schrock, Snodgrass, & Sugiyama, 2020). Such heavy reliance on mutual aid implies selection for incentivising one's mates, friends, allies and fellow group members to render assistance in times of hunger, incapacitation, or interpersonal conflict (Sugiyama, 2004). Because being positively valued by fellow group members would have led to being helped more and exploited less, how our ancestors were valued by those around them would have had considerable impact on their reproductive success (von Rueden & Jaeggi, 2016).

Research indicates that when new information comes to light that reveals an individual to be less socially valuable to others than previously supposed (e.g. lacking in skills, selfish, diseased, of reduced social status), audiences react by attaching less weight to that person's welfare (e.g. see Delton & Robertson, 2016; Sell, 2005; Lim, 2012; Sznycer, 2010; Ermer, 2007; Sparrowe, 2020). Over evolutionary time, individuals devalued in this manner would have incurred fitness costs by, for example, being avoided, shunned, denied help and/or ostracised more often (Kurzban & Leary, 2001; Hales et al., 2016). For our ancestors, the adaptive problem of being devalued would therefore have made the difference between a long, successful life and an early, possibly violent demise.

## The information threat theory of shame

The need to avoid the costs resulting from devaluation would have selected for regulatory adaptations to minimise the spread of negative information about the self and the costs associated with any ensuing devaluation that resulted from it (Gilbert, 1997; Tooby & Cosmides, 2008; Sznycer et al., 2016; Fessler, 1999; Weisfeld & Dillon, 2012). Over the millennia, those individuals who better attracted goodwill and avoided the indifference, censure and wrath of potential aid-givers would have more frequently survived and reproduced. This *information threat theory of shame* therefore posits that shame is an adaptation that evolved to defend against information-triggered devaluation (Landers, Sznycer & Al Shawaf, 2022; Sznycer, 2010; Sznycer et al., 2016; Sznycer, Sell, & Lieberman, 2021; Sznycer, Cosmides, & Tooby, 2017; Robertson et al., 2018; see also Gilbert, 1997; Fessler, 1999; Weisfeld & Dillon, 2012; Baumeister & Tice, 1990; Schlenker & Leary, 1982). According to this account, the shame system is an adaptation designed to coordinate psychology, physiology and behaviour to: (a) inhibit actions likely to yield more costs from social devaluation than the benefits said actions would yield; (b) limit the spread of potentially discrediting information about the self; (c) minimise the degree and scope of any social devaluation that does occur; and, if devaluation occurs, (d) motivate actions geared toward mitigating its costs.

In line with this account, experimentally manipulating (potential or actual) devaluation reliably elicits shame (Smith et al., 2002; Dickerson et al., 2008; Robertson et al., 2018). Being devalued – disliked, excluded, tortured, oppressed – by others elicits shame even when individuals know they haven't done anything wrong (Robertson et al., 2018; Shapiro, 2003; Levi, 1989). The true elicitor of shame, therefore, appears to be neither objective wrongdoing nor one's causal attributions for one's wrongdoing (Tracy & Robins, 2004), but rather the *threat or actuality of social devaluation* (Robertson et al., 2018). Wrongdoing so often triggers shame because wrongdoing reliably predicts the threat of being devalued, but wrongdoing is neither necessary nor sufficient for triggering shame.

The information threat theory proposes that the shame system operates by incorporating both an invariant architecture *and* open parameters. This is because the best-bet response to counter devaluation depends on the internal and external environments in which the shame adaptation finds itself: you may or may not have enough physical strength or high-status allies to resist an attack against you; you may or may not have personal qualities that could compensate for your devalued trait; by the time you hear the sound of footsteps approaching, you may or may not have enough time to sneak out of the room before you're caught red-handed. These open parameters take variable, dynamically updated inputs – e.g. an audience is present but not expressing displeasure (Dickerson et al., 2008); ‘I am well-connected’ (Sznycer et al., 2012); ‘the audience has weapons’ – and feeds them into the invariant architecture. Utilising specialised concepts (e.g. AUDIENCE; Sznycer, 2010), the invariant architecture then specifies the decision logic that matches sub-classes of inputs to sub-classes of behavioural outputs (e.g. terminate action, give excuse) to minimise the devaluative threat (Sznycer, 2010; Lukaszewski et al., 2020; Sznycer & Cohen, 2021; see Table 1).

**Table 1.** Hypothesised outputs of the shame system as a function of its mode of operation

Mode of operation	Outputs
**Prospectively**: discrediting behaviours or qualities may potentially be emitted (but the individual knows they have not yet been)	Precautionary measures to limit the broadcast of discrediting cues.
**Prospectively**: cues of discrediting behaviours or qualities are known to have been emitted but inferred to not have reached others’ minds	Little overt behaviour. ‘Hiding in plain sight’. ‘Playing dumb’
**Prospectively**: cues of discrediting behaviours or qualities are known to have been emitted but the individual is uncertain whether they have reached others’ minds	(Stealthy) search for cues of one's social value among the audience. Attempting to learn who knows what. Anxiety about how the situation will resolve
**Reactively:** cues of discrediting behaviours or qualities are inferred to have reached others’ minds, and the individual is uncertain whether the audience has or will devalue him	
**Reactively:** cues of discrediting behaviours or qualities are inferred to have reached others’ minds, but the individual infers that the audience has not and will not devalue him	Precautionary measures to prevent leakage of information from the current audience to others who might devalue the focal individual
**Reactively:** cues of discrediting behaviours, or qualities are inferred to have reached others’ minds, and the individual infers the audience has or will devalue him	Behavioral inhibition, delivery of benefits to the audience, appeasement, submission, infliction of costs, or combinations thereof

Adapted from Sznycer (2010: 165).

Differences in the expression of the shame system across situations, individuals and populations can thus be interpreted as differences in the distributions of relevant inputs fed into the open parameters. For example, the cost of devaluation can be mitigated by forming new alliances. Therefore, the information threat theory predicts that individual differences in shame-proneness will depend in part on perceptions of the ease with which one can form new relationships (i.e. ‘relational mobility’; see Yuki et al., 2007) to compensate for lost or damaged relationships. Testing this prediction, Sznycer et al. (2012) compared the shame responses of Western participants with those of East-Asian participants – traditionally thought to be more shame prone (Benedict, 1946) – and found that this ‘cultural’ difference was partially mediated by East-Asian participants’ perception of lower relational mobility (Sznycer et al., 2012).

Additionally, the information threat theory predicts that individuals with highly valued (or feared) traits (e.g. resourcefulness, physical attractiveness, being well-connected) will be less prone to shame all else equal, because their superior position can be leveraged to (a) impose or threaten to impose more costs on others to incentivise less devaluation or prevent it altogether (Sell, Tooby, & Cosmides, 2009) and (b) allow them to better weather the consequences of devaluation when they are devalued. As predicted, such individuals are less prone to shame (Sznycer et al., 2012). Similarly, variation in audience composition should affect the degree of shame activation: a high-status audience, for example, can impose greater costs on others (and withhold greater benefits), and so being devalued by such an audience should be more shame-provoking than being devalued by a low-status audience, all else equal. In line with this, Garland and Brown (1972) found that in a task where longer singing time increased participant payment, teenage girls who evaluated their singing as poor sang longer when their audience was described as made up of ‘poor singers’ than when their audience was described as made up of ‘excellent singers’ (Garland & Brown, 1972). Likewise, Jackson and Latané (1981) found that participants reported more nervousness at the prospect of singing in front of an audience of graduate students and professors from a music school than at the prospect of singing in front of tone-deaf undergraduates. In line with the information threat theory, the shame people experience seems well calibrated to the specifics of the threat posed by devaluation.

## A novel prediction derived from the information threat theory

To minimise the threat of devaluation aptly, the shame system must operate not only reactively, when one knows the audience is expressing or has expressed devaluative responses, but also prospectively, in advance of any action likely to bring about social devaluation (Sznycer et al., 2016; Leary, 2015; Sommerville, Schmidt, Yun, & Burns, 2013; see also Fehr & Gächter, 2000). To activate prospectively, the shame system needs accurate estimates of the degree to which fellow group members devalue a given act or personal characteristic. In short, the shame system needs to predict the precise magnitude of likely devaluation for a given act or trait, before any actual devaluation takes place, in order to guide decisions cost-effectively. According to the information threat theory, anticipatory feelings of shame internally transmit precise information about the predicted magnitude of audience devaluation one would incur were one to engage in an act disfavoured by the audience, weighted by the odds of being discovered by the audience (Sznycer et al., 2016, see also Crockett, Siegel, Kurth-Nelson, Dayan, & Dolan, 2017). This internal signal helps avoid the dual errors of prospective shame under-activation, whereby the individual mounts an insufficient response to prevent or diminish devaluation (and thus incurs excessive costs from others’ devaluation), and prospective shame over-activation, whereby the individual experiences so much shame that they refrain from taking actions that would yield more direct benefits than the costs they would incur from that devaluation.

**Figure 1.**
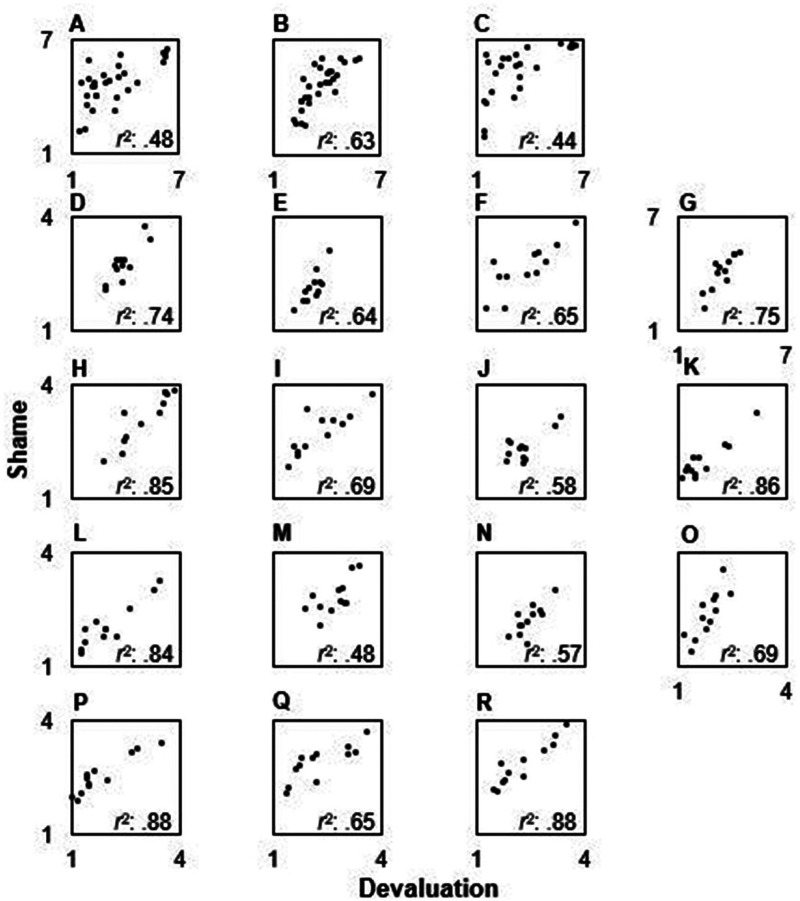
Shame tracks devaluation. The intensity of anticipatory shame tracks the intensity of audience devaluation. Scatterplots A–C: data from three mass societies (adapted from Sznycer et al., 2016). The stimuli were a set of brief hypothetical scenarios describing socially devalued actions and personal characteristics. The scenarios were phrased either from the perspective of the focal individual (e.g. ‘You are not generous with others’; *shame* condition) or from the perspective of an observer vis-à-vis the focal individual (e.g. ‘He is not generous with others’; *audience* condition; between-subjects design). For each scenario, participants rated either their feeling of shame if they took those actions or had those characteristics (*shame*), or the degree to which they would negatively view the individual in the scenarios if the individual took those actions or had those characteristics (*audience*). Each point represents the mean shame rating and mean devaluation rating of one scenario. Data from (number of scenarios): A, US (29); B, India (29); C, Israel (24). Scatterplots D–R, data from 15 small-scale societies (adapted from Sznycer et al., 2018). Same experimental design, but with a set of 12 scenarios that were different from the ones used in Sznycer et al. (2016). D, Cotopaxi, Ecuador; E, Morona-Santiago, Ecuador; F, Coquimbo, Chile; G, Drâa-Tafilalet, Morocco; H, Enugu, Nigeria; I, Chalkidiki, Greece; J, Ikland, Uganda; K, Le Morne, Mauritius; L, La Gaulette, Mauritius; M, Dhading, Nepal; N, Tuva, Russia; O, Khövsgöl, Mongolia; P, Shaanxi, China; Q, Farming Communities, Japan; R, Fishing Communities, Japan. In all cases, shame ratings and devaluation ratings were given by different participants. Effect sizes: *r*^2^ linear.

This reasoning led to empirical tests of the hypotheses that the shame system is designed to (a) accurately forecast the magnitude of audience devaluation on an act-by-act basis, and (b) generate an internal signal of anticipatory shame in proportion to that forecast. Across three different cultures (the US, India and Israel), participants were presented with a set of socially devalued acts or traits (e.g. theft, sexual infidelity, poor table manners, lack of ambition) and were asked to rate in a between-subjects design, for each act or trait, either the intensity of shame they would feel if the act or trait were true of them or how negatively they would view another individual if the act or trait were true of that other individual (a measure of devaluation). In all three countries, the intensity of anticipatory shame closely tracked the magnitude of devaluation expressed by local audiences (Figure 1a–c). Furthermore, shame within each country tracked the magnitude of devaluation reported in the other two countries, suggesting universality in both the structure and content of shame (Sznycer et al., 2016; see also Durkee, Lukaszewski, & Buss, 2019; Cohen, Chun, & Sznycer, 2020). Follow-up studies demonstrated that this close association is specific to shame: other negatively valenced emotions such as sadness and anxiety did *not* track audience devaluation (Sznycer et al., 2016). Moreover, further follow-up studies conducted in 15 traditional small-scale societies around the world again found close associations between shame and audience devaluation: shame in each population closely tracked both the devaluation reported locally and the devaluation reported in each of the other 14 populations. Notably, measures of linguistic similarity, religious similarity, and geographic proximity – three common measures of cultural distance – each failed to account for the strength of between-community associations between shame and devaluation (Sznycer et al., 2018; Figure 1, d–r).

## The information threat theory can account for many known features of shame

The hypothesis that shame's adaptive function is to mitigate the threat of devaluation can explain many published findings on shame. For instance, being devalued causes people pain (MacDonald & Leary, 2005; Eisenberger, 2012); people generally avoid actions that could exacerbate devaluation (De Hooge, Breugelmans, & Zeelenberg, 2008; Fehr & Gächter, 2000); among students and depressed patients, shame proneness correlates with perceptions of low status and submissive behaviour (Gilbert, 2000a); people tend to hide reputationally damaging information (Rockenbach & Milinski, 2011; Sznycer, Schniter, Tooby, & Cosmides, 2015); and when other people discover reputationally damaging information about them, shamed individuals withdraw (Leach & Cidam, 2015), appease those who know it (Keltner, Young, & Buswell, 1997) and apologise to them (Schniter, Sheremeta, & Sznycer, 2013). Furthermore, shame is exacerbated when failing at easy (vs. difficult) tasks – expected if low competence elicits more devaluation and warrants more shame (Lewis et al., 1992). Some evidence suggests that shame increases with severity of disease, perhaps because severe diseases are impairing or communicable, and individuals suffering from such diseases are less capable of aiding others or defending their own interests (Leary, Rapp, Herbst, Exum, & Feldman, 1998; Homayoon et al., 2020).

The information threat theory can explain the link between being victimised and feeling shame – an ethical and scientific puzzle, since victims are often victimised through no fault of their own. Some evidence indeed suggests such a link. For instance, being a victim of torture (Shapiro, 2003), marital violence (Andrews & Brewin, 1990), rape (Notman & Nadelson, 1976) and physical and sexual abuse during childhood (Andrews & Hunter, 1997) elicit shame and self-blame. Both literary and sociological data (Hovannisian, 1986; Levi, 1989; Totten, 2009) indicate that shame is prevalent in victims of wanton subjugation, where ‘on a rational plane, there should not have been much to be ashamed of’ (Levi, 1989, p. 77). Yet while this link between victimisation and shame may seem odd or anomalous if shame is triggered as a result of attributing negative outcomes to the self (Tangney, Wagner, & Gramzow, 1992) or violating a norm (Fessler, 1999), from the perspective of the information threat theory, victimisation can meet the input conditions of devaluation: after all, being victimised often results from being devalued by others, and the inability to resist victimisation may indicate low personal formidability or status – personal characteristics that meet the input conditions of the shame programme.

When it comes to behavioural outputs, shame can produce a wide and seemingly paradoxical array of actions. For instance, shame can generate both enhanced cooperativeness and hostile tactics, such as shifting blame to victims, scapegoating third parties and threatening or mobilising aggression (Leach & Cidam, 2015; Fessler, 2001; Scheff, 1987; Elison et al., 2014; Zhu et al., 2019), making amends (De Hooge et al., 2008; De Hooge, Breugelmans, Wagemans, & Zeelenberg, 2018; Sznycer et al., 2015), avoiding others (Barrett, Zahn-Waxler, & Cole, 1993), feeling depressed (Tangney et al., 1992; Cheung, Gilbert, & Irons, 2004) and attempting to improve oneself (De Hooge et al., 2010). Such heterogeneous tactics can interfere with each other if deployed concurrently (e.g. cooperation and aggression) but have the capacity to minimise the threat of devaluation if deployed in the right situations or sequences.

## Forms and functions of the shame display

**Figure 2.**
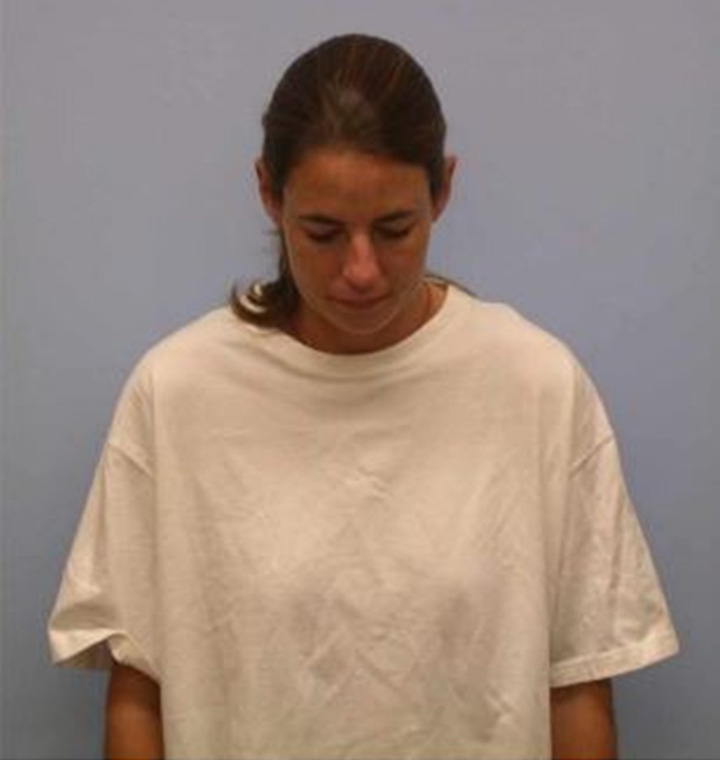
Prototypical shame display. Reprinted from Martens et al. (2012), copyright (2022) by Taylor & Francis. Reprinted with permission.

One hallmark of shame is a characteristic display that includes slumped posture, gaze aversion, downward head tilt (Fessler, 1999; Keltner et al. 1997; Tracy, Robins, & Schriber, 2009; see Figure 2) and behavioural inhibition, including reduced speech (Gilbert, 1998; Price & Sloman, 1987; Fessler, 1999). The shame display is produced when people experience personal failure (Tracy & Matsumoto, 2008; Witkower, Mercadante, & Tracy, 2020), and shame displayers are correspondingly perceived by observers as having suffered a reduction in their social status (Shariff, Tracy, & Markusoff, 2012). The shame display develops reliably; it is produced by young children (Barrett, 1998; Lewis et al., 1992; Stipek et al., 1992) and congenitally blind adults (Tracy & Matsumoto, 2008). The shame display also affords teachable moments: people infer from expressions of shame what fellow group members devalue and correspondingly downgrade their inclination to perform those devalued actions or activities (Schaumberg & Skowronek, 2022).

The human shame display appears to be phylogenetically ancient; it resembles the displays of submission produced by non-human species in aggressive contests over dominance (Martens, Tracy, & Shariff, 2012; Tracy, Robins, & Schriber, 2009). Behavioral homologues or analogues of human shame occur widely across species (Gilbert, 2000b): in barbary macaques (Deag, 1977), rhesus monkeys (Møller, Harlow, & Mitchell, 1968), chimpanzees (Nishida, 1983), lemurids (Pereira & Kappeler, 1997), mice, guinea-pigs and golden hamsters (Grant & Mackintosh, 1963), laboratory rats (Blanchard & Blanchard, 1977), canids (Darwin, 1872/2009; Fox, 1969), pigs (McGlone, 1985), lizards (Carpenter, 1962), black-crowned night herons (Noble, Wurm, & Schmidt, 1938), fish (Grosenick, Clement, & Fernald, 2007; Lorenz, 1966) and stone crabs (Sinclair, 1977).

Compared with non-human animals, status in humans depends less heavily on fighting ability or readiness to submit to the powerful (Gilbert, 1997; Fessler, 1999) and more heavily on the individual's ability and willingness to confer benefits (Durkee, Lukaszewski, & Buss, 2020; Eisenbruch, Grillot, Maestripieri & Roney, 2016; von Rueden, Gurven, Kaplan, 2008) and to abide by the coordinated values of fellow group members (Gilbert, 1997; Fessler, 1999). This difference has led some researchers to question the adaptive value of the shame display in modern humans: the gaze avoidance and general submissive stance delivered by the shame display may have been useful for signaling submission and deterring further attacks in our ancestral past, but may now no longer serve such a function in our modern, coordinated, prestige-driven societies (Gilbert & McGuire, 1998; Gilbert, 1997; see also Curtis & Miller, 1986). From an adaptationist standpoint, however, one might ask: are there features in the human shame display that seem improbably well-designed to solve an adaptive problem – here, defend against the threat of devaluation – relative to what chance would produce (Williams, 1966)? An affirmative answer to this question seems quite plausible. The human shame display seems well-engineered to cost-effectively address two distinct sub-problems related to information-triggered devaluation: (a) mollifying observers when one has been identified as a transgressor; and (b) reducing observers’ ability to identify one as a transgressor in the first place.

## Mollifying observers

Gaze avoidance is a hallmark of the shame display, but people generally judge gaze-avoiders unfavourably (Larsen & Schackelford, 1996). This raises the question of why a component of the shame system – putatively designed to counter devaluation – would produce actions that increase devaluation. This puzzle may be solved, however, when considering that the relevant comparison is not between observer evaluations of gaze seekers and gaze avoiders generally, but rather between observer evaluations of gaze seekers and gaze avoiders whom observers know have committed disreputable behaviour. When evaluated against this more relevant standard, gaze avoidance appears to have *positive* (less negative) effects for gaze avoiders: indeed, when an individual commits a moral infraction of which observers are aware, individuals who subsequently gaze-avoid are judged *more favourably* than those who gaze-seek. And audiences’ responses toward individuals known to have transgressed are more forgiving when the transgressor produces the gaze-avoiding shame display than when the transgressor fails to produce gaze-avoidance (De Jong, 1999; Keltner, Young, & Buswell, 1997; Semin & Manstead, 1982). For example, Keltner and colleagues (1997) found that audience members were more sympathetic toward students whom they were told had failed a presentation when those students exhibited the shame display rather than an embarrassment display (Keltner et al., 1997). And in a follow-up experiment, it was found that observers judged a convicted criminal to be less guilty and assigned them less prison time when criminals displayed shame or embarrassment compared with neutral expressions. Similarly, Giner-Sorolla, Castano, Espinosa, and Brown (2008) found that when told of a CEO who verbally apologised on behalf of his company for a chemical spill, audiences were more accepting of the apology when he expressed shame than when he expressed guilt or no emotion (Giner-Sorolla et al., 2008). Further, in experiments asking participants to assign penalties to fictitious sex offenders, Proeve and Howells (2006) found that audiences were more lenient when told the offenders expressed shame than when they expressed sadness or remorse (Proeve & Howells, 2006). These findings demonstrate the effect of the gaze-avoidant shame display to mollify the angry reactions of observers, and suggest, contra mismatch arguments, that shame-driven gaze avoidance need not be a vestigial feature on its way to being selected out (see Sznycer, 2010).

The mollifying effect of gaze avoidance (and the general submissive stance) on audiences has been interpreted as appeasement (Keltner et al., 1997) – the evolutionarily conserved inhibition of another's aggression by signaling that one withdraws from a contest (Fessler, 2007; Sloman, Price, Gilbert, Gardner, 1994; Weisfeld & Dillon, 2012). However, positive evaluations of gaze avoidance have also been observed in contexts in which there is no apparent transgression. For example, gaze-avoidance is judged less favourably than gaze-seeking when the target is physically attractive or expressing happiness, but gaze-avoidance is judged *more* favourably when the target is physically unattractive or expressing disgust (Main, DeBruine, Little, & Jones, 2010; Rall, Greenspan, & Neidich, 1984). The latter effects may still fall under the rubric of appeasement, however, if gaze avoidance signals the pre-emptive rejection of a claim for attention or social valuation perceived as undeserved. Nevertheless, more research is needed to affirm the appeasement hypothesis.

## The invisibility hypothesis

While a sizeable literature deals with the appeasing function of the shame display, no research we are aware of has elaborated on the possibility that the shame ‘display’ might serve another function: evasion from identification (and thus from devaluation). We use quotation marks around ‘display’ here because according to this hypothesis, the characteristic constellation of shame actions functions not to elicit a forgiving response from audiences, but to evade recognition and evaluation by the audience. That is, in some contexts the shame ‘display’ may function as camouflage rather than as a signal (Smith & Harper, 2003). In what follows, we describe the logic behind this idea (what we term *the invisibility hypothesis*), suggest ways the shame system could realise this function, and offer questions that future research could address.

If shame functions to diminish costs owing to devaluation as the information threat theory suggests, then one powerful way to accomplish that goal would be to prevent identification or recognition of the transgressor in the first place. Or, to paraphrase Sun Tzu (2010), the most effective form of appeasement for one's transgressions is the one not required. If caught committing a harmful, immoral, or disgraceful act, appeasing those who discover you might be prudent. However, you'd be even better served to evade detection and identification entirely, thus saving yourself the cost of appeasement (itself an admission of reduced status or social value) and any ensuing reduction in status or esteem. As Zhu and colleagues have shown (2019), people prefer not to appease: when feeling shame, people tolerate being treated poorly by others when others know why they feel ashamed but angrily protest that same poor treatment when it can be portrayed credibly as undeserved – that is, when people know others are in the dark about their transgression (Zhu et al., 2019).

The view of the shame ‘display’ as a means to achieve invisibility can be found in Darwin's *Expression of the Emotions in Man and Animals*:

Under a keen sense of shame there is a strong desire for concealment. We turn away the whole body, more especially the face, which we endeavour in some manner to hide. An ashamed person can hardly endure to meet the gaze of those present, so that he almost invariably casts down his eyes or looks askant. (1872/2009: 340)

Incidentally, we note that the hunger for invisibility – to act free of the threat of others’ devaluation and aggression, to gain information about others unmolested by reputational concerns, to snoop around without being regarded as a snoop (Rockenbach & Milinsky, 2011; Dana, Weber & Kuang, 2007) – appears to be a human universal. Allusions to this desire can be found across time and cultures, from Plato's *Ring of Gyges*, Tolkien's *The One Ring*, Rowling's *Cloak of Invisibility*, Wells’ *Invisible Man* and Marvel Comics’ *Invisible Woman*, to the Daoist texts on goddess Jiutian Xuannü and Persian mythology's *Khrafstra*.

**Figure 3.**
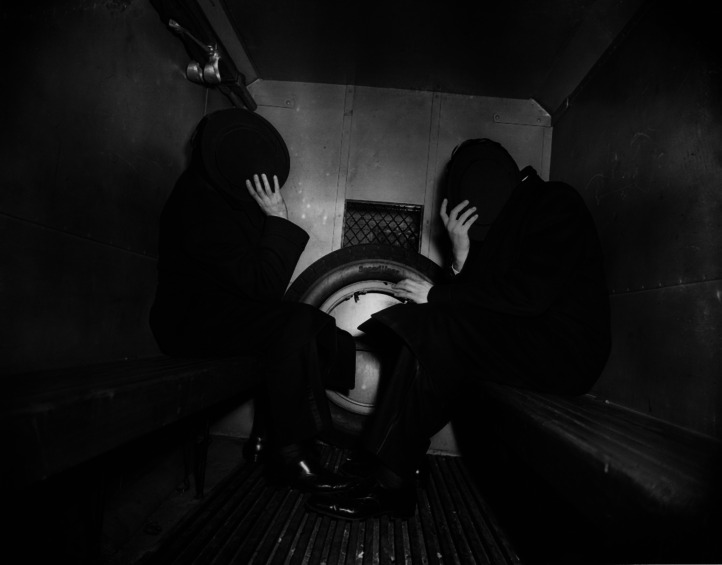
Concealment through total occlusion of the face. A. Fellig (1944). ‘In The Paddy Wagon’ (photograph by Weegee/International Center of Photography). Via Getty Images. https://www.gettyimages.com/detail/news-photo/premium-rates-apply-not-to-be-used-for-postcards-news-photo/2716706?adppopup=true

The idea that the shame system may be designed to evade devaluation by rendering one invisible (Tooby, personal communication) may seem odd or hyperbolic, but shame can render people invisible *to audiences* in the literal sense that shame operates preemptively and anticipatorily (Sznycer et al., 2016, 2018), leading people to interrupt their disgraceful actions, hide, flee or destroy incriminating evidence when they perceive an audience looming. Thus, the shame system can motivate behaviour that makes invisible one's disgraceful act. However, when evading observers is impractical, the static shame ‘display’, or camouflage, may still confer some measure of invisibility, albeit to a lesser (and less literal) degree. This camouflage may be effective at interfering with the audience's attention, face-processing, identification, recognition, encoding and other information-gathering pre-requisites for devaluing wrongdoers or ‘undesirables’ (i.e. via down-regulation of welfare tradeoff ratio; see Tooby, Cosmides, Sell, Lieberman, & Sznycer, 2008; Delton & Robertson, 2016) and thus at interfering with the behaviours that result from that devaluation (e.g. disassociation, condemnation, banishment). The static shame ‘display’-as-camouflage may confer its producer a measure of invisibility both when occluding one's face with a top hat (see Figure 3) and in less extreme cases such as in the prototypical shame ‘display’ (Figure 2).

The shame system can produce invisibility in various ways: leading one to forgo actions that may result in being devalued, interrupting one's actions when detection by others is imminent, hiding, fleeing, equivocating, deceiving, justifying one's actions, and concocting other ruses for concealing oneself in plain sight. How might the static shame ‘display’, or camouflage, confer invisibility? We suggest two possible ways.

### Interfering with identification

One way in which the shame camouflage could confer invisibility is by disrupting observers’ ability to identify the ashamed individual. Data exist which are consistent with this possibility. For example, observers are quicker to find faces in an array (Von Grünau & Anston, 1995) and to categorise such faces (Macrae, Hood, Milne, Rowe, & Mason, 2002) when those faces have direct gaze (vs. gaze averted). Similarly, faces with gazes trained at an observer (vs. with gazes averted as in the shame display) capture more of observers’ attention (Baron-Cohen, 1995; Senju & Hasegawa, 2005; Frischen, Bayless, & Tipper, 2007). Importantly, direct eye gaze, relative to gaze aversion, enhances unconscious processing of faces (Stein, Senju, Peelen, & Sterzer, 2011). Additionally, Young and colleagues (2014) have shown with a composite face task that neither participant accuracy nor reaction time differed across aligned and misaligned conditions for faces with averted gaze, suggesting gaze aversion interferes with configural facial processing (Young, Slepian, Wilson, & Hugenburg, 2014). Further, because the meeting of gazes creates common knowledge among gazers, gaze avoidance may also function to hamper the generation of common knowledge between the transgressor and audience regarding the transgressor's complicity (Thomas, DeScioli, & Pinker, 2018), something that may subsequently allow transgressors to plausibly deny or dispute the facts (or others’ interpretation of the facts) of their disgraceful act (see Pinker, Nowak, & Lee, 2008).

### Interfering with memory encoding

Another way in which the shame camouflage could confer invisibility is by disrupting the observers’ ability to encode in memory the ashamed individual as the perpetrator of a disgraceful act. In line with this possibility, faces with direct (vs. averted) gaze are better remembered by observers (Hood, Macrae, Cole-Davies, & Dias, 2003; Mason et al., 2004; Smith, Hood, & Hector, 2006); this effect seems to influence facial memory at both the encoding and retrieval phases (Hood et al., 2003). While such results are consistent with the invisibility hypothesis, more research is needed to examine how emotional expression influences facial memory. Work by Nakashima and colleagues (2012), for example, found an interactive effect of emotional expression and eye gaze on facial memory, such that angry faces were remembered less well when gaze was averted than directed, while happy faces were unaffected by gaze direction (Nakashima, Langton, & Yoshikawa, 2012). Future work could explore whether the shame pose – compared with more neutral expressions – is remembered less well and/or interacts with eye gaze to influence facial memory.

While there are both anecdotal and systematic data consistent with the invisibility hypothesis, no research has yet directly tested whether the characteristic syndrome of shame actions functions (at least in part) to render shamed individuals invisible to observers – that is, to interfere with observers’ ability to identify or recall ashamed individuals and thus to devalue them. In what follows, we offer a series of questions to help guide future research on the invisibility hypothesis.

How quickly do observers identify targets who adopt the characteristic static shame pose (Figure 2) vs. a neutral pose/expression or other combinations of poses and expressions (e.g. combat readiness)? For example, Hansen and Hansen (1988) found that angry faces are more quickly picked out of a crowd of neutral or happy faces than happy or neutral faces are picked out of a crowd of angry faces (Hansen & Hansen, 1988). Might the shame pose be less quickly picked out of a crowd of neutral or alternative expressions compared with non-shame emotional expressions (e.g. anger)? How quickly do observers pick out shame poses vs. neutral poses or other expressions within an array of distractors? What process or processes contribute to a possible reduction in identification speed? One candidate process is attention: how much attention does the shame pose capture relative to neutral or other emotional expressions? We know from previous research that averted gaze captures less attention than direct gaze (Baron-Cohen, 1995; Senju & Hasegawa, 2005), but might other features of the prototypical shame pose capture less attention than their non-shame alternatives as well? How does the shame pose influence memory? Are individuals who exhibit the shame pose recalled more infrequently than individuals who exhibit other emotional or neutral expressions? What are the corresponding rates of memory decay for individuals exhibiting these emotional expressions? And what process or processes contribute to any differences in memory decay?

The hypothesised invisibility effect of the shame pose on both memory and identification is likely to interact with observer knowledge: how would learning the identity of the culprit who committed the shame-inducing act alter observer identification of and/or memory for individuals who exhibit the shame pose relative to other emotional expressions? Learning that a shameful event has occurred but lacking knowledge of the culprit, or lacking knowledge of any shameful event occurring in first place, may also uniquely affect observer identification and/or memory for individuals exhibiting the shame pose relative to other emotional expressions. Precisely because the shame display may also function as a signal of appeasement, learning that a shameful event has occurred, but lacking knowledge of the culprit, may *increase* observer identification of and/or memory for individuals exhibiting the shame pose relative to other expressions. Indeed, in reactive mode, the shame system may produce a shame display designed to call attention to one's submission, coupled with characteristic verbal and nonverbal actions drawing attention to one's acceptance of lower status (e.g. saying ‘I'm sorry’). In prospective mode, however, before observers know that a transgression has occurred, the shame pose may reduce observer identification and/or memory compared with other kinds of emotional expressions. To corroborate the invisibility hypothesis, research must demonstrate reduced observer ability to identify and/or recall individuals exhibiting the shame pose relative to other emotional expressions given at least one level of observer knowledge.

According to the invisibility hypothesis, invisibility is conferred by adaptive design. This assumes that net benefits from invisibility would have accrued over deep time on average to those who adopted the shame pose in the right place at the right time *in the kinds of high-coresidence, tightly knit ecologies in which our ancestors evolved*. Therefore, an important test of the invisibility hypothesis is whether *known community members* (vs. the strangers generally used as stimuli in research) attain invisibility dividends when they adopt the shame pose. The attainment of invisibility by known community members seems less probable, because observers possess a wealth of background knowledge that can aid in identifying familiar transgressors (e.g. height, body shape, dress), and more is at stake for observers when identifying actual wrongdoers in their community compared to when identifying the fictional wrongdoing of strangers. Yet this high evidentiary bar needs to be met for the invisibility hypothesis to show viability.

Answers to these and related questions will corroborate or falsify the invisibility hypothesis.

## Concluding remarks

An adaptationist approach to shame paints a unique picture of this emotion: rather than a maladaptive source of hostility and psychopathology, shame is a messenger, one that defends the individual by transmitting devaluation-relevant information to an array of countermeasures against devaluation. The information threat theory can explain many known facts about shame and generate novel predictions. Some of these have received broad support across cultures (Sznycer et al., 2016, 2018; Durkee et al., 2019; Cohen et al., 2020; Sznycer & Cohen, 2021). Others, like the invisibility hypothesis, remain to be tested.

## References

[ref2] Andrews, B., & Brewin, C. R. (1990). Attributions of blame for marital violence: A study of antecedents and consequences. Journal of Marriage and the Family, 52, 757–767.

[ref3] Andrews, B., & Hunter, E. (1997). Shame, early abuse, and course of depression in a clinical sample: A preliminary study. Cognition & Emotion, 11(4), 373–381.

[ref4] Baron-Cohen, S. (1995). The eye direction detector (EDD) and the shared attention mechanism (SAM): Two cases for evolutionary psychology. Presented at the *Society for Research in Child Development Conference*, New Orleans, March 1993; the British Psychological Society, Welsh Branch, *‘Faces*’ *Conference*, University of Wales College of Cardiff, September 1993; and the British Society for the Philosophy of Science’ *Roots of Joint Reference*’ *Conference*, University of Bristol, November 1993. Lawrence Erlbaum.

[ref5] Barrett, K. C. (1998). A functionalist perspective to the development of emotions. In M. F. Mascolo & S. Griffin (Eds.), What develops in emotional development? (pp. 109–133). Plenum.

[ref6] Barrett, K. C., Zahn-Waxler, C., & Cole, P. M. (1993). Avoiders vs. *amenders: Implications for the investigation of guilt and shame during toddlerhood?* Cognition & Emotion, 7(6), 481–505.

[ref7] Baumeister, R. F. & Tice, D. M. (1990). Point–counterpoints: Anxiety and social exclusion. Journal of Social and Clinical Psychology, 9, 165–195.

[ref8] Benedict, R. (1946). The chrysanthemum and the sword. Houghton Mifflin.

[ref9] Blanchard, R. J., & Blanchard, D. C. (1977). Aggressive behavior in the rat. Behavioral Biology, 21(2), 197–224. 562152 10.1016/s0091-6773(77)90308-x

[ref10] Brown, D. E. (1991). Human universals. Temple University Press.

[ref11] Burger, O., Baudisch, A., & Vaupel, J. W. (2012). Human mortality improvement in evolutionary context. Proceedings of the National Academy of Science, USA, 109(44), 18210–18214. 10.1073/pnas.1215627109 PMC3497824 23071331

[ref12] Carpenter, C. C. (1962). Patterns of behavior in two Oklahoma lizards. American Midland Naturalist, 67(1), 132–151.

[ref13] Cheung, M. P., Gilbert, P., & Irons, C. (2004). An exploration of shame, social rank and rumination in relation to depression. Personality and Individual Differences, 36(5), 1143–1153.

[ref14] Clutton-Brock, T. (2009). Cooperation between non-kin in animal societies. Nature 462, 51–57. 10.1038/nature08366 19890322

[ref15] Cohen, A. S., Chun, R., & Sznycer, D. (2020). Do pride and shame track the evaluative psychology of audiences? Preregistered replications of Sznycer et al. (2016, 2017). Royal Society Open Science, 7, 191922. 32537196 10.1098/rsos.191922 PMC7277259

[ref16] Crockett, M. J., Siegel, J. Z., Kurth-Nelson, Z., Dayan, P., & Dolan, R. J. (2017). Moral transgressions corrupt neural representations of value. Nature Neuroscience, 20(6), 879–885. 28459442 10.1038/nn.4557 PMC5462090

[ref17] Curtis, R. C., & Miller, K. (1986). Believing another likes or dislikes you: Behaviors making the beliefs come true. Journal of Personality and Social Psychology, 51(2), 284.

[ref18] Dana, J., Weber, R. A., & Kuang, J. X. (2007). Exploiting moral wiggle room: Experiments demonstrating an illusory preference for fairness. Economic Theory, 33(1), 67–80.

[ref19] Darwin, C. R. (1872/2009). The expression of emotions in man and animals. Cambridge University Press.

[ref20] Deag, J. M. (1977). Aggression and submission in monkey societies. Animal Behaviour, 25, 465–474.

[ref21] De Hooge, I. E., Breugelmans, S. M., Wagemans, F. M., & Zeelenberg, M. (2018). The social side of shame: Approach versus withdrawal. Cognition and Emotion, 32(8), 1671–1677. 29303420 10.1080/02699931.2017.1422696

[ref22] De Hooge, I. E., Breugelmans, S. M., & Zeelenberg, M. (2008). Not so ugly after all: When shame acts as a commitment device. Journal of Personal and Social Psychology, 95, 933–943. 10.1037/a0011991 18808269

[ref23] De Hooge, I.E., Zeelenberg, M., & Breugelmans, S. (2010). Restore and protect motivations following shame. Cognition and Emotion, 24, 111–127.

[ref24] De Jong, P. J. (1999). Communicative and remedial effects of social blushing. Journal of Nonverbal Behavior, 23(3), 197–217.

[ref25] Delton, A. W., & Robertson, T. E. (2016). How the mind makes welfare tradeoffs: Evolution, computation, and emotion. Current Opinion in Psychology, 7, 12–16.

[ref26] Dickerson, S. S., Mycek, P. J., & Zaldivar, F. (2008). Negative social evaluation, but not mere social presence, elicits cortisol responses to a laboratory stressor task. Health Psychology, 27(1), 116. 10.1037/0278-6133.27.1.116 18230022

[ref27] Dimberg, U., & Öhman, A. (1983). The effects of directional facial cues on electrodermal conditioning to facial stimuli. Psychophysiology, 20(2), 160– 167. 6844515 10.1111/j.1469-8986.1983.tb03282.x

[ref28] Durkee, P. K., Lukaszewski, A. W., & Buss, D. M. (2019). Pride and shame: Key components of a culturally universal status management system. Evolution and Human Behavior, 40(5), 470–478.

[ref29] Durkee, P. K., Lukaszewski, A. W., & Buss, D. M. (2020). Psychological foundations of human status allocation. Proceedings of the National Academy of Sciences, 117(35), 21235–21241. 10.1073/pnas.2006148117 PMC7474695 32817486

[ref30] Eisenberger N. I. (2012). The pain of social disconnection: Examining the shared neural underpinnings of physical and social pain. Nature Reviews Neuroscience, 13(6), 421–434. 22551663 10.1038/nrn3231

[ref31] Eisenbruch, A. B., Grillot, R. L., Maestripieri, D., & Roney, J. R. (2016). Evidence of partner choice heuristics in a one-shot bargaining game. Evolution and Human Behavior, 37(6), 429–439.

[ref32] Elison, J., Garofalo, C., & Velotti, P. (2014). Shame and aggression: Theoretical considerations. Aggression and Violent Behavior, 19, 447–453

[ref33] Ermer, E. R. (2007). Coalitional support and the regulation of welfare tradeoff ratios. University of California, Santa Barbara.

[ref34] Fehr, E., & Gächter, S. (2000). Cooperation and punishment in public goods experiments. American Economics Review, 90(4), 980–994.

[ref35] Fessler, D. M. T. (2001). Emotions and cost/benefit assessment: The role of shame and self-esteem in risk taking. In R. Selten & G. Gigerenzer (Eds.), Bounded Rationality: The Adaptive Toolbox (pp. 191–214). MIT University Press.

[ref36] Fessler, D. M. T. (2004). Shame in two cultures: Implications for evolutionary approaches. Journal of Cognition and Culture, 4(2), 207–262.

[ref37] Fessler, D. M. T. (1999). Toward an understanding of the universality of second order emotions (A. L. Hinton, Ed., pp. 75–116). Cambridge University Press.

[ref38] Fessler, D. M. T. (2007). From appeasement to conformity: Evolutionary and cultural perspectives on shame, competition, and cooperation (J. L. Tracy, R. W. Robins, & J. P. Tangney, Eds., pp. 174–193). Guilford Press.

[ref39] Fox, M. W. (1969). The anatomy of aggression and its ritualization in Canidae: A developmental and comparative study. Behaviour, 35, 242–258.

[ref40] Frischen, A., Bayliss, A. P., & Tipper, S. P. (2007). Gaze cueing of attention: Visual attention, social cognition, and individual differences. Psychological Bulletin, 133(4), 694. 17592962 10.1037/0033-2909.133.4.694 PMC1950440

[ref41] Garland, H., & Brown, B. R. (1972). Face-saving as affected by subjects’ sex, audiences’ sex and audience expertise. *Sociometry,* 35(2), 280–289. 5033657

[ref42] Gilbert, P. (1997). The evolution of social attractiveness and its role in shame, humiliation, guilt and therapy. British Journal of Medical Psychology, 70, 113–147. 9210990 10.1111/j.2044-8341.1997.tb01893.x

[ref43] Gilbert, P. (1998). What is shame? Some core issues and controversies. In P. Gilbert & B. Andrews (Eds.), Shame: Interpersonal behavior, psychopathology, and culture (pp. 3–38). Oxford University Press.

[ref44] Gilbert, P. (2000a). The relationship of shame, social anxiety and depression: The role of the evaluation of social rank. Clinical Psychology & Psychotherapy: An International Journal of Theory & Practice, 7(3), 174–189.

[ref45] Gilbert, P. (2000b). Social mentalities: Internal ‘social’ conflicts and the role of inner warmth and compassion in cognitive therapy. In P. Gilbert & K. G. Bailey (Eds.), Genes on the couch: Explorations in evolutionary psychotherapy (pp. 118–150). Psychology Press.

[ref46] Gilbert, P., & McGuire, M. T. (1998). Shame, status, and social roles: Psychobiology and evolution. In P. Gilbert & B. Andrews (Eds.), Shame: Interpersonal behavior, psychopathology, and culture (pp. 99–125). Oxford University Press.

[ref47] Giner-Sorolla, R., Castano, E., Espinosa, P., & Brown, R. (2008). Shame expressions reduce the recipient's insult from outgroup reparations. Journal of Experimental Social Psychology, 44(3), 519–526.

[ref48] Grant, E. C., & Mackintosh, J. H. (1963). A comparison of the social postures of some common laboratory rodents. Behaviour, 21, 246–259.

[ref49] Grosenick, L., Clement, T. S., & Fernald, R. D. (2007). Fish can infer social rank by observation alone. Nature, 445(7126), 429–432. 17251980 10.1038/nature05511

[ref50] Hales, A. H., Kassner, M. P., Williams, K. D., & Graziano, W. G. (2016). Disagreeableness as a cause and consequence of ostracism. Personality and Social Psychology Bulletin, 42, 782–797. 27044246 10.1177/0146167216643933

[ref51] Hansen, C. H., & Hansen, R. D. (1988). Finding the face in the crowd: an anger superiority effect. Journal of Personality and Social Psychology, 54(6), 917. 10.1037//0022-3514.54.6.917 3397866

[ref52] Hill, K., & Hurtado, A. M. (1996). Ache life history: The ecology and demography of a foraging people. De Gruyter.

[ref53] Homayoon, D., Hiebler-Ragger, M., Zenker, M., Weger, W., Unterrainer, H., & Aberer, E. (2020). Relationship between skin shame, psychological distress and quality of life in patients with psoriasis: A pilot study. Acta Dermato-Venereologica, 100(14), 00015555-3563. 10.2340/00015555-3563 PMC9199924 32556357

[ref54] Hood, B. M., Macrae, C. N., Cole-Davies, V., & Dias, M. (2003). Eye remember you: The effects of gaze direction on face recognition in children and adults. Developmental Science, 6(1), 67–71.

[ref55] Hovannisian, R. G. (Ed.). (1986). *The Armenian genocide in perspective.* Transaction Publishers.

[ref56] Jackson, J. M., & Latané, B. (1981). All alone in front of all those people: Stage fright as a function of number and type of co-performers and audience. Journal of Personality and Social Psychology, 40(1), 73.

[ref57] Kaplan, H., & Hill, K. (1985). Food sharing among ache foragers: Tests of explanatory hypotheses. Current Anthropology, 26, 223–239.

[ref58] Keeley, L. H. (1997). War before civilization. Oxford University Press.

[ref59] Keltner, D., & Anderson, C. (2000). Saving face for Darwin. Current Directions in Psychological Science, 9(6), 187–192.

[ref60] Keltner, D., Young, R. C., & Buswell, B. N. (1997). Appeasement in human emotion, social practice, and personality. Aggressive Behavior, 23, 359–374.

[ref61] Kurzban, R., & Leary, M. R. (2001). Evolutionary origins of stigmatization: The functions of social exclusion. Psychology Bulletin, 127, 187–20860 10.1037/0033-2909.127.2.187 11316010

[ref62] Landers, M., Sznycer, D., & Al-Shawaf, L. (2022). Shame. In L. Al-Shawaf & T. K. Shackelford (Eds.), The Oxford handbook of evolution and the emotions. Oxford University Press.

[ref64] Larsen, R. J., & Shackelford, T. K. (1996). Gaze avoidance: Personality and social judgments of people who avoid direct face-to-face contact. Personality and Individual Differences, 21(6), 907–917. 10.1016/S0191-8869(96)00148-1

[ref65] Leach, C. W., & Cidam, A. (2015). When is shame linked to constructive approach orientation? A meta-analysis. Journal of Personal and Socical Psychology, 109(6), 983–1002.27. 10.1037/pspa0000037 26641074

[ref66] Leary, M. R. (2015). Emotional responses to interpersonal rejection. Dialogues in Clinical neuroscience, 7, 435. 10.31887/DCNS.2015.17.4/mleary PMC4734881 26869844

[ref67] Leary, M. R., Rapp, S. R., Herbst, K. C., Exum, M. L., & Feldman, S. R. (1998). Interpersonal concerns and psychological difficulties of psoriasis patients: Effects of disease severity and fear of negative evaluation. Health Psychology, 17(6), 530. 10.1037//0278-6133.17.6.530 9848803

[ref68] Levi, P. (1989). The drowned and the saved. Vintage.

[ref69] Lewis, M., Alessandri, S. M., & Sullivan, M. W. (1992). Differences in shame and pride as a function of children's gender and task difficulty. Child Development, 63(3), 630–638. 10.2307/1131351 1600827

[ref70] Lim, J. (2012). Welfare tradeoff ratios and emotions: Psychological foundations of human reciprocity. University of California, Santa Barbara.

[ref71] Lorenz, K. (1966). On aggression. Harcourt Brace & World.

[ref72] Lukaszewski, A. W., Lewis, D. M. G., Durkee, P. K., Sell, A. N., Sznycer, D., & Buss, D. M. (2020). An adaptationist framework for personality science. European Journal of Personality, 34, 1151–1174.

[ref73] MacDonald, G., & Leary, M. R. (2005). Why does social exclusion hurt? The relationship between social and physical pain. Psychological Bulletin, 131(2), 202. 10.1037/0033-2909.131.2.202 15740417

[ref74] Macrae, C. N., Hood, B. M., Milne, A. B., Rowe, A. C., & Mason, M. F. (2002). Are you looking at me? Eye gaze and person perception. Psychological Science, 13(5), 460–464. 12219814 10.1111/1467-9280.00481

[ref75] Main, J. C., DeBruine, L. M., Little, A., & Jones, B. C. (2010). Interactions among the effects of head orientation, emotional expression, and physical attractiveness on face preferences. Perception, 39(1), 62–71. 10.1068/p6503 20301847

[ref76] Martens, J. P., Tracy, J. L., & Shariff, A. F. (2012). Status signals: Adaptive benefits of displaying and observing the nonverbal expressions of pride and shame. Cognition & Emotion, 26(3), 390–406. 22471848 10.1080/02699931.2011.645281

[ref77] Mason, M. F., Hood, B. M., & Macrae, C. N. (2004). Look into my eyes: Gaze direction and person memory. Memory, 12, 637–643. 15615320 10.1080/09658210344000152

[ref78] McGlone, J. J. (1985). A quantitative ethogram of aggressive and submissive behaviors in recently regrouped pigs. Journal of Animal Science, 61(3), 556–566. 4066526

[ref79] Møller, G. W., Harlow, H. F., & Mitchell, G. D. (1968). Factors affecting agonistic communication in rhesus monkeys (*Macaca mulatta*). Behaviour, 31, 339–357. 4973591 10.1163/156853968x00324

[ref80] Mosquera, P. M., Manstead, A. S., & Fischer, A. H. (2002). The role of honour concerns in emotional reactions to offences. Cognition & Emotion, 16(1), 143–163.

[ref81] Nakashima, S. F., Langton, S. R., & Yoshikawa, S. (2012). The effect of facial expression and gaze direction on memory for unfamiliar faces. Cognition & Emotion, 26(7), 1316–1325. 22077759 10.1080/02699931.2011.619734

[ref82] Nishida, T. (1983). Alpha status and agonistic alliance in wild chimpanzees (*Pan troglodytes schweinfurthii*). Primates, 24(3), 318–336.

[ref83] Noble, G. K., Wurm, M., & Schmidt, A. (1938). Social behavior of the black-crowned night heron. The Auk, 55(1), 7–40.

[ref84] Notman, M. T., & Nadelson, C. C.. (1976). The rape victim: Psychodynamic considerations. *The American Journal of Psychiatry*, 133(4), 408–413. 1267039 10.1176/ajp.133.4.408

[ref85] Öhman, A., & Dimberg, U. (1978). Facial expressions as conditioned stimuli for electrodermal responses: A case of ‘preparedness’? Journal of Personality and Social Psychology, 36(11), 1251–1258. 745035 10.1037//0022-3514.36.11.1251

[ref86] Pereira, M. E., & Kappeler, P. M. (1997). Divergent systems of agonistic behaviour in lemurid primates. Behaviour, 134, 225–274.

[ref87] Pinker, S., Nowak, M. A., & Lee, J. J. (2008). The logic of indirect speech. Proceedings of the National Academy of Sciences, 105(3), 833–838. 10.1073/pnas.0707192105 PMC2242675 18199841

[ref88] Price, J. S., & Sloman, L. (1987). Depression as yielding behavior: An animal model based on Schjelderup-Ebbe's pecking order. Ethology and Sociobiology, 8, 85–98.

[ref89] Proeve, M. J., & Howells, K. (2006). Effects of remorse and shame and criminal justice experience on judgements about a sex offender. Psychology, Crime & Law, 12(2), 145–161.

[ref90] Rall, M., Greenspan, A., & Neidich, E. (1984). Reactions to eye contact initiated by physically attractive and unattractive men and women. Social Behavior and Personality: An International Journal, 12(1), 103–110.

[ref91] Robertson, T. E., Sznycer, D., Delton, A. W., Tooby, J., & Cosmides, L. (2018). The true trigger of shame: Social devaluation is sufficient, wrongdoing is unnecessary. Evolution and Human Behavior, 39, 566–573.

[ref92] Rockenbach, B., & Milinski, M. (2011). To qualify as a social partner, humans hide severe punishment, although their observed cooperativeness is decisive. Proceedings of the National Academy of Science, USA, 108, 18307–18312. 10.1073/pnas.1108996108 PMC3215000 21987800

[ref93] Schaumberg, R. L., & Skowronek, S. E. (2022). Shame broadcasts social norms: The positive social effects of shame on norm acquisition and normative behavior. Psychological Science, 09567976221075303. 10.1177/09567976221075303 35797281

[ref94] Scheff, T. J. (1987). The shame–rage spiral: A case study of an interminable quarrel. In H. B. Lewis (Ed.), The role of shame in symptom formation (pp. 109–49). Erlbaum.

[ref95] Scherer, K. R., & Wallbott, H. G. (1994). Evidence for universality and cultural variation of differential emotion response patterning. Journal of Personality and Social Psychology, 66(2), 310–328. 8195988 10.1037//0022-3514.66.2.310

[ref96] Schlenker, B. R., & Leary, M. R. (1982). Social anxiety and self-presentation: A conceptualization model. Psychology Bulletin, 92, 641–669. 10.1037/0033-2909.92.3.641 7156261

[ref97] Schniter, E., Sheremeta, R. M., & Sznycer, D. (2013). Building and rebuilding trust with promises and apologies. Journal of Economic Behavior and Organization, 94, 242–256.

[ref98] Schrock, J. M., Snodgrass, J. J., & Sugiyama, L. S. (2020). Lassitude: The emotion of being sick. Evolution and Human Behavior, 41, 44–57.

[ref99] Sell, A. (2005). Regulating welfare tradeoff ratios: Three tests of an evolutionary-computational model of human anger. University of California, Santa Barbara.

[ref100] Sell, A., Tooby, J., & Cosmides, L. (2009). Formidability and the logic of human anger. Proceedings of the National Academy of Science, USA, 106(35), 15073–15078. 10.1073/pnas.0904312106 PMC2736438 19666613

[ref101] Semin, G. R., & Manstead, A. S. R. (1982). The social implications of embarrassment displays and restitution behavior. European Journal of Social Psychology, 12, 367–377.

[ref102] Senju, A., & Hasegawa, T. (2005). Direct gaze captures visuospatial attention. Visual Cognition, 12, 127–144

[ref103] Shapiro, D. (2003). The tortured, not the torturers, are ashamed. Social Research: An International Quarterly, 70(4), 1131–1148.

[ref104] Shariff, A. F., Tracy, J. L., & Markusoff, J. L. (2012). (Implicitly) judging a book by its cover: The power of pride and shame expressions in shaping judgments of social status. Personality and Social Psychology Bulletin, 38(9), 1178–1193. 10.1177/0146167212446834 22611053

[ref105] Shweder, R. (2003). Toward a deep cultural psychology of shame. Social Research: An International Quarterly, 70(4), 1109–1129.

[ref106] Sinclair, M. E. (1977). Agonistic behaviour of the stone crab, *Menippe mercenaria* (Say). Animal Behaviour, 25, 193–207.

[ref107] Sloman, L., Price, J. S., Gilbert, P., & Gardner, R. (1994). Adaptive function of depression: Psychotherapeutic implications. American Journal of Psychotherapy, 48, 401–416. 7992871 10.1176/appi.psychotherapy.1994.48.3.401

[ref108] Smith, A. D., Hood, B. M., & Hector, K. (2006). Eye remember you two: Gaze direction modulates face recognition in a developmental study. Developmental Science, 9(5), 465–472. 16911448 10.1111/j.1467-7687.2006.00513.x

[ref109] Smith, J. M., & Harper, D. (2003). Animal signals. Oxford University Press.

[ref110] Smith, R. H., Webster, J. M., Parrott, W. G., & Eyre, H. L. (2002). The role of public exposure in moral and nonmoral shame and guilt. Journal of Personal and Social Psychology, 83, 138–15971. 12088123

[ref111] Sommerville, J. A., Schmidt, M. F., Yun, J. E., & Burns, M. (2013). The development of fairness expectations and prosocial behavior in the second year of life. Infancy, 18(1), 40–66.

[ref112] Sparrowe, R. T. (2020). LMX and welfare trade-off ratios: An evolutionary perspective on leader-member relations. The Leadership Quarterly, 31(2), 101271.

[ref113] Stein, T., Senju, A., Peelen, M. V., & Sterzer, P. (2011). Eye contact facilitates awareness of faces during interocular suppression. Cognition, 119(2), 307–311. 21316650 10.1016/j.cognition.2011.01.008 PMC3796336

[ref114] Stipek, D., Recchia, S., & McClintic, S. (1992). Self-evaluation in young children. Monographs of the Society for Research in Child Development, 57(1), 100. 10.2307/1166190 1560797

[ref115] Sugiyama, L. S. (2004). Illness, injury, and disability among Shiwiar forager–horticulturists: Implications of health-risk buffering for the evolution of human life history. American Journal of Physical Anthropology, 123, 371–389. 15022365 10.1002/ajpa.10325

[ref116] Sznycer, D. (2010). Cognitive adaptations for calibrating welfare tradeoff motivations, with special reference to the emotion of shame. Doctoral dissertation (University of California, Santa Barbara).

[ref117] Sznycer, D., & Cohen, A. S. (2021). Are emotions natural kinds after all? Rethinking the issue of response coherence. Evolutionary Psychology. 10.1177/14747049211016009. PMC10355299 34060370

[ref118] Sznycer, D., Cosmides, L., & Tooby, J. (2017). Adaptationism carves emotions at their functional joints. *Invited commentary in* Psychological Inquiry, 28(1), 56–62.

[ref119] Sznycer, D., Schniter, E., Tooby, J., & Cosmides, L. (2015). Regulatory adaptations for delivering information: The case of confession. Evolutionary Human Behavior, 36, 44–51. 10.1016/j.evolhumbehav.2014.08.008 PMC4313746 25663798

[ref120] Sznycer, D., Sell, A., & Lieberman, D. (2021). Forms and functions of the social emotions. Current Directions in Psychological Science, 30(4), 292–299.

[ref121] Sznycer, D., Takemura, K., Delton, A. W., Sato, K., Robertson, T., Cosmides, L., & Tooby, J. (2012). Cross-cultural differences and similarities in proneness to shame: An adaptationist and ecological approach. Evolutionary Psychology, 10(2), 147470491201000213. 10.1177/147470491201000213 PMC3604996 22947644

[ref122] Sznycer, D., Tooby, J., Cosmides, L., Porat, R., Shalvi, S., & Halperin, E. (2016). Shame closely tracks the threat of devaluation by others, even across cultures. Proceedings of the National Academy of Sciences, USA, 113, 2625–2630. 10.1073/pnas.1514699113 PMC4790975 26903649

[ref123] Sznycer, D., Xygalatas, D., Agey, E., Alami, S., An, X.-F., Ananyeva, K. I., … Tooby, J. (2018). Cross-cultural invariances in the architecture of shame. Proceedings of the National Academy of Sciences, USA, 115(39), 9702–9707. 10.1073/pnas.1805016115 PMC6166838 30201711

[ref124] Tangney, J. P., Wagner, P., & Gramzow, R. (1992). Proneness to shame, proneness to guilt, and psychopathology. Journal of Abnormal Psychology, 101(3), 469. 10.1037//0021-843x.101.3.469 1500604

[ref125] Thomas, K. A., DeScioli, P., & Pinker, S. (2018). Common knowledge, coordination, and the logic of self-conscious emotions. Evolutionary Human Behavior, 39(2), 179–190.

[ref126] Tooby, J., & Cosmides, L. (1992). The psychological foundations of culture. In J. H. Barkow, L. Cosmides, & J. Tooby (Eds.), The adapted mind: Evolutionary psychology and the generation of culture (pp. 19–136). Oxford University Press.

[ref127] Tooby, J. & Cosmides, L. (2008). The evolutionary psychology of the emotions and their relationship to internal regulatory variables. In M. Lewis, J. M. Haviland-Jones & L. F. Barrett (Eds.), Handbook of emotions (3rd ed., pp. 114–137). Guilford.

[ref128] Tooby, J. & Cosmides, L. (2015). The theoretical foundations of evolutionary psychology. In Buss, D. M. (Ed.), The handbook of evolutionary psychology (2nd ed., Vol. 1: Foundations, pp. 3–87). John Wiley & Sons.

[ref129] Tooby, J., Cosmides, L., Sell, A., Lieberman, D., & Sznycer, D. (2008). Internal regulatory variables and the design of human motivation: A computational and evolutionary approach. Handbook of Approach and Avoidance Motivation, 15, 251.

[ref130] Totten, S. (2009). Plight and fate of women during and following genocide. Transaction.

[ref131] Tracy, J. L., & Matsumoto, D. (2008). The spontaneous expression of pride and shame: Evidence for biologically innate nonverbal displays. Proceedings of the National Academy of Sciences, USA, 105(33), 11655–11660. 10.1073/pnas.0802686105 PMC2575323 18695237

[ref132] Tracy, J. L., & Robins, R. W. (2004). Putting the self into self-conscious emotions: A theoretical model. Psychological Inquiry, 15, 103–125.

[ref133] Tracy, J. L., Robins, R. W., & Schriber, R. A. (2009). Development of a FACS-verified set of basic and self-conscious emotion expressions. Emotion, 9(4), 554–559. 10.1037/a0015766 19653779

[ref134] Tzu, S. (2010). The art of war. Capstone.

[ref135] Von Grünau, M., & Anston, C. (1995). The detection of gaze direction: A stare-in-the-crowd effect. Perception, 24(11), 1297–1313. 8643334 10.1068/p241297

[ref136] von Rueden, C., Gurven, M., & Kaplan, H. (2008). The multiple dimensions of male social status in an Amazonian society. Evolution and Human Behavior, 29(6), 402–415. 10.1016/j.evolhumbehav.2008.05.001 19884954 PMC2598750

[ref137] von Rueden, C., & Jaeggi, A. V. (2016). Men's status and reproductive success in 33 nonindustrial societies: Effects of subsistence, marriage system, and reproductive strategy. Proceedings of the National Academy of Science, USA, 113(39), 10824–10829. 10.1073/pnas.1606800113 PMC5047206 27601650

[ref138] Weisfeld, G. E., & Dillon, L. M. (2012). Applying the dominance hierarchy model to pride and shame, and related behaviors. Journal of Evolutionary Psychology, 10, 15–41.

[ref139] Williams, G. C. (1966). Natural selection, the costs of reproduction, and a refinement of Lack's principle. The American Naturalist, 100(916), 687–690.

[ref140] Witkower, Z., Mercadante, E. J., & Tracy, J. L. (2020). How affect shapes status: Distinct emotional experiences and expressions facilitate social hierarchy navigation. Current Opinion in Psychology, 33, 18–22. 10.1016/j.copsyc.2019.06.006 31336192

[ref141] Young, S. G., Slepian, M. L., Wilson, J. P., & Hugenberg, K. (2014). Averted eye-gaze disrupts configural face encoding. Journal of Experimental Social Psychology, 53, 94–99.

[ref142] Yuki, M., Schug, J., Horikawa, H., Takemura, K., Sato, K., Yokota, K., … Kamaya, K. (2007). Development of a scale to measure perceptions of relational mobility in society. (CERSS Working Paper 75). Sapporo, Japan: Center for Experimental Research in Social Sciences, Hokkaido University.

[ref143] Zhu, R., Xu, Z., Tang, H., Liu, J., Wang, H., An, Y., Mai, X., & Liu, C. (2019). The effect of shame on anger at others: Awareness of the emotion-causing events matters. Cognition and Emotion, 33(4), 696–708. 10.1080/02699931.2018.1489782 29932822

